# Early Physical Therapist Interventions for Patients With COVID-19 in the Acute Care Hospital: A Case Report Series

**DOI:** 10.1093/ptj/pzaa194

**Published:** 2020-10-19

**Authors:** Sabrina Eggmann, Angela Kindler, Andrea Perren, Natalie Ott, Frauke Johannes, Rahel Vollenweider, Théophile Balma, Claire Bennett, Ivo Neto Silva, Stephan M Jakob

**Affiliations:** Department of Physiotherapy, Insel Group, Inselspital, Bern University Hospital, Bern, Switzerland; Department of Physiotherapy, Insel Group, Inselspital, Bern University Hospital, Bern, Switzerland; Department of Physiotherapy, Insel Group, Inselspital, Bern University Hospital, Bern, Switzerland; Institute of Therapies and Rehabilitation, Kantonsspital Winterthur, Winterthur, Switzerland; Department of Physiotherapy and Occupational Therapy, University Hospital Zurich, Zurich, Switzerland; Department of Physiotherapy and Occupational Therapy, University Hospital Zurich, Zurich, Switzerland; Department of Surgery and Anesthesia, Cardio-Respiratory Physiotherapy, Lausanne University Hospital, Lausanne, Switzerland; Intensive Care Unit, Department of Acute Care Medicine, Geneva University Hospitals (HUG), Geneva, Switzerland; Intensive Care Unit, Department of Acute Care Medicine, Geneva University Hospitals (HUG), Geneva, Switzerland; Department of Intensive Care Medicine, Inselspital, Bern University Hospital, University of Bern, Bern, Switzerland

**Keywords:** COVID-19, Early Mobilization, Critical Illness, Physiotherapy, Dysphagia, Delirium, Early Ambulation, Critical Care, Case Report

## Abstract

**Objective:**

The aim of this case series was to describe the experience of Swiss physical therapists in the treatment of patients with COVID-19 during their acute care hospital stay and to discuss challenges and potential strategies in the clinical management of these patients.

**Methods:**

We report 11 cases of patients with COVID-19 from 5 Swiss hospitals that illustrate the various indications for physical therapy, clinical challenges, potential treatment methods, and short-term response to treatment.

**Results:**

Physical therapists actively treated patients with COVID-19 on wards and in the intensive care unit. Interventions ranged from patient education, to prone positioning, to early mobilization and respiratory therapy. Patients were often unstable with quick exacerbation of symptoms and a slow and fluctuant recovery. Additionally, many patients who were critically ill developed severe weakness, postextubation dysphagia, weaning failure, or presented with anxiety or delirium. In this setting, physical therapy was challenging and required specialized and individualized therapeutic strategies. Most patients adopted the proposed treatment strategies, and lung function and physical strength improved over time.

**Conclusion:**

Physical therapists clearly have a role in the COVID-19 pandemic. Based on our experience in Switzerland, we recommend that physical therapists routinely screen and assess patients for respiratory symptoms and exercise tolerance on acute wards. Treatment of patients who are critically ill should start as soon as possible to limit further sequelae. More research is needed for awake prone positioning and early breathing exercises as well as post-COVID rehabilitation.

**Impact:**

To date, there are few data on the physical therapist management of patients with COVID-19. This article is among the first to describe the role of physical therapists in the complex pandemic environment and to describe the potential treatment strategies for countering the various challenges in the treatment of these patients.

## Background and Purpose

On March 11, 2020, the World Health Organization called the worldwide outbreak of COVID-19 disease caused by the novel SARS coronavirus 2 (SARS-CoV-2) a pandemic.^1^ At that time, Switzerland counted 1161 cases and 7 fatalities.^2^ Subsequently, the virus spread rapidly through the Swiss population resulting in one of the highest incidence rates of infections per capita worldwide.^3^

Common clinical symptoms of COVID-19 include fever (80%), cough (63%), fatigue (46%), and expectoration (42%).^4^ Respiratory failure including acute respiratory distress syndrome (ARDS) has been reported in approximately 20% of cases.^4^ In northern Italy, which is very close to the Swiss border, approximately 9% of infected patients were admitted to an intensive care unit (ICU) for mechanical ventilation.^5^ Physical therapists’ activities in the treatment of patients with COVID-19 include early exercise and mobilization, tracheostomy management, and the implementation of prone positioning in the ICU.^6^^,^^7^

In Switzerland, physical therapists commonly provide early rehabilitation in the acute care hospital, which includes early mobilization, respiratory management, and functional exercises.^8^^,^^9^ Physical therapists were therefore involved very early in the care of patients with COVID-19 and obliged to meet an increased demand for therapy services by the beginning of March. This was met by increasing service presence up to 24 hours for 7 days a week and by recruiting physical therapists with a cardiorespiratory background from other teams (eg, pediatrics) as suggested by Thomas et al.^6^ The main goals of physical therapy were first, to provide respiratory care and intensive rehabilitation to facilitate functional recovery and hospital discharge; and second, to assist in prone positioning in the ICU to ensure adequate skin care and proper joint positioning. Additionally, physical therapists were active in extraclinical activities such as training, mentoring, and research.

The aim of this case series is to describe our experience in the treatment of patients with COVID-19 during their acute hospital stay and to discuss treatment responses and challenges. We describe 11 selected cases of COVID-19 from 5 Swiss hospitals, illustrate potential physical therapist interventions, demonstrate the large variability of this illness, and discuss future recommendations for clinical practice and research. The local ethics committees waived the need for approval. All survivors provided written informed consent.

## Case Descriptions and Outcomes

**Figure 1:**
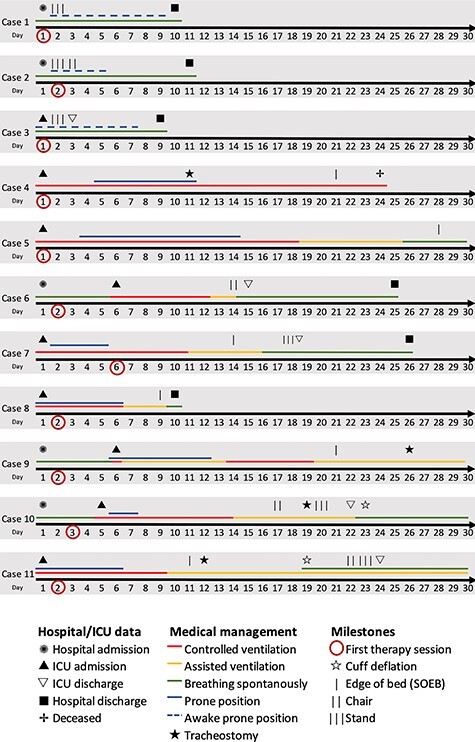
Hospital timeline. ICU = intensive care unit; SOEB = sitting on the edge-of-bed.

Settings, participant selection, and data collection are described in the Supplementary Material. Cases were retrospectively selected to represent the spectrum of symptoms and interventions. All patients tested positive for SARS-CoV-2. Their characteristics and physical therapist interventions are summarized in the Table. Figure 1 illustrates the timeline of cases’ medical management and ICU and hospital data as well as achieved milestones.

**Table:** Patient Characteristics and Physical Therapist Interventions*^a^*

Case	Body Functions (Main Findings)	Main Therapy Goals	Total Sessions, Frequency, Duration, Number of Physical Therapists	Physical Therapy Interventions	Therapy Equipment	IMS at Hospital Admission/Discharge	Adverse Events*^b^*	Relevant Comorbidities	Severity of Illness, Medical Management	LOS Hospital/ICU, Discharge Destination
1	Dry cough, dyspnea, mild general weakness	Improved oxygenation, decreased cough frequency, increased strength and endurance capacity	8 sessions, once daily (except for Sundays), 25-60 min with 1 PT	Respiratory therapy (ACBT, MITF, inhalation), side and prone positioning, mobilization, strength training (squats, calf raises), walk training	No specialized equipment	8/10	None	Arterial hypertension, ex-smoker	SOFA 2 (respiration at hospital admission), symptomatic therapy, supplemental oxygen	10 d/– Rehabilitation
2	Generally deconditioned, severely breathless during minor activities	Alleviation of dyspnea, improved oxygenation, decreased anxiety, improved independent mobility	10 sessions, once daily, 30-40 min with 1 PT	Patient education, deep-breathing exercises, positioning (forward sitting, prone), strength training (sit-to-stand, step-ups on stair, calf raises), reconditioning	No specialized equipment	8/10	None	None	Symptomatic therapy and monitoring, supplemental oxygen	11 d/– Home
3	Normal muscle strength (MRC-SS 60/60), normal ROM, dyspnea	Improved oxygenation	9 sessions, 1-2 times per day, 30 min with 1 PT	Self-proning, respiratory therapy (ACBT), early mobilization, walking exercises, squats	No specialized equipment	10/10	None	Arterial hypertension, obstructive sleep apnea syndrome	APACHE II: 9, symptomatic therapy and monitoring, supplemental oxygen	9 d/3 d Home
4	Frontal decubitus, severe muscle loss, normal ROM	Prevention of secondary complications	21 sessions, once daily; pROM: 15 min; side-edge: 25 min; proning: 60 min with 1 PT	pROM, proning, side-edge position	No specialized equipment	0/1	None	Several chronic cardiac and neurological comorbidities	APACHE II: 33, mechanical ventilation, proning, tracheostomy, vasopressors, sedation, neuromuscular blocking agents, CRRT, symptomatic therapy	24 d/24 d Died
5	Bronchial mucus, weak cough, dyspnea, respiratory insufficiency	Airway clearance, maintain adequate gas exchange, relieve dyspnea	Total number of sessions unknown, 2-6 times per day, 15-60 min with 1-2 PTs	Respiratory therapy (manual compressions, nasal rinsing), proning, pROM, assistive exercises, mobilization, strength training (squats), on spot walking exercises	No specialized equipment	1/(3 at ICU discharge)*^b^*	None	Arterial hypertension, obesity, obstructive sleep apnea syndrome, ex-smoker	Mechanical ventilation, proning, vasopressors, sedation, neuromuscular blocking agents, inhaled nitric oxide, antivirals (lopinavir-ritonavir), symptomatic therapy	53 d/40 d*^c^*
6	Severe muscle loss, severe weakness, oral/pharyngeal sensibility disorder and severe weakness, delirium	Prevention from aspiration, increased muscle strength and function	22 sessions, 1-3 times daily, 30-60 min, with 1 PT	pROM, positioning, breathing therapy, mobilization, dysphagia therapy, strength training	No specialized equipment	3/10	None	Arterial hypertension	SOFA score: 11 (at ICU admission), mechanical ventilation, intermittent dialysis, sedation, vasopressor, symptomatic therapy	25 d/10 d Rehabilitation
7	Muscle weakness (MRC-SS 40/60), moderate physical functioning (PFIT-s 9/12), normal ROM, dysphagia	Prevention of secondary complications, increase physical and muscle function, increase alertness, weaning from mechanical ventilation	19 sessions, 1-2 times per day, 30-60 min. 1-3 PTs per session: eg, 1 for pROM, 3 for SOEB (day 14), 2 for transfer from bed to chair (day 18)	pROM, proning, mobilization, standing, transferring from bed to chair, respiratory care	No specialized equipment	0/9	None	Asthma, hypertension, diabetes, obesity	APACHE II: 27; SAPS II score: 44 (at ICU admission), mechanical ventilation, supplemental oxygen, inhaled nitric oxide, proning, vasopressors, sedation, neuromuscular blocking agents, symptomatic therapy	26 d/18 d Rehabilitation
8	Severe muscle loss, normal ROM, dysphagia, delirium, anxiety	Prevention of secondary complications, increased function	16 session, 1-2 times per day, pROM: 15 min, proning: 60 min, rehab: 30 min with 1 PT	pROM, proning, mobilization, dysphagia therapy, respiratory therapy	In-bed cycle ergometer	0/3	None	None	APACHE II: 25, mechanical ventilation, proning, vasopressors, sedation, neuromuscular blocking agents, CRRT, symptomatic therapy	10 d/10 d Transferred to other hospital
9	Severe muscle loss, severe cardiac insufficiency, reduced alertness, poor physical function	Increased function, increased alertness, weaning from mechanical ventilation	43 sessions, 1-2 times per day, 30-60 min with 1 PT	pROM, proning, side-edge position, mobilization, perception training, dysphagia therapy	No specialized equipment	0/3 (at time of writing)*^b^*	None	Arterial hypertension, diabetes	SOFA score: 12 (at ICU admission), mechanical ventilation, proning, tracheostomy, vasopressors, sedation, neuromuscular blocking agents, CRRT, symptomatic therapy	35 d/27 d*^c^*
10	Mild muscle weakness (MRC-SS 48/60), slightly reduced physical functioning (PFIT-s 10/12), inspiratory muscle weakness, dysphagia, tracheostomy	Prevention of secondary complications, increased function, increased alertness, weaning from mechanical ventilation	13 sessions, 1-2 times per day, 30-60 min. 1 PT for pROM, 2 PTs for SOEB and sit-to-stand exercise	pROM, proning, mobilization, standing, chair, in-bed cycling, respiratory care	In-bed cycle ergometer	10/(5 at ICU discharge)*^b^*	None	None	SAPS II: 27 (at ICU admission), mechanical ventilation, supplemental oxygen, proning, tracheostomy, vasopressors, sedation, neuromuscular blocking agents, symptomatic therapy	>35 d/18 d*^c^*
11	Severe muscle loss, delirium, normal ROM, dysphagia, tracheostomy	Prevention of secondary complications, increased muscle strength, increased function	52 sessions, 1-2 times per day, pROM: 15 min, proning: 60 min, rehab: 45 min with 1 PT	pROM, proning, respiratory therapy, mobilization, in-bed cycling, dysphagia therapy	In-bed cycle ergometer, speaking valve, threshold inspiratory muscle trainer, standing and mobilization aids	0/5	None	Coronary vascular disease with arterial hypertension, smoker	APACHE II: 30, mechanical ventilation, proning, tracheostomy, vasopressors, sedation, neuromuscular blocking agents	39 d/24 d Rehabilitation

^*^a^*^ACBT = active cycle of breathing technique; APACHE = Acute Physiology and Chronic Health Evaluation (assessed within 24 hours of ICU admission); CRRT = continuous renal replacement therapy; ICU = intensive care unit; IMS = ICU mobility scale (minimum 0; maximum 10); LOS = length of stay; MITF = maximal inspiration and stretching in side position; MRC-SS = Medical Research Council sum score (minimum 0; maximum 60); PFIT-s = physical function ICU test—scored (minimum 0; maximum 12);^17^ (p)ROM = (passive) range of motion; PT = physical therapist; SAPS = Simplified Acute Physiology Score; SOEB = sitting on the edge-of-bed; SOFA = Sequential Organ Failure Assessment.

^*^b^*^Defined as a device dislocation, fall, cardiac arrest, death, or any other serious adverse event during physical therapy.

^*^c^*^Has not been discharged at the time of writing.

### Case 1: Respiratory Instability

This 60-year-old male was hospitalized due to moderate ARDS from COVID-19 with symptoms of fever, dry cough, and dyspnea. We encountered several difficulties during physical therapy on the acute ward. First, any change of position or deep breathing triggered coughing attacks that induced oxygen desaturation and dyspnea. To avoid rapid deterioration and respiratory failure, we instructed and performed position changes very slowly and step-by-step. In this way, a position change to the 135° prone position (Suppl. Fig. 1) took around 30 minutes. This approach was well tolerated and increased oxygen saturation, for example**,** on day 5 with 6 L/min of oxygen from 93% to 97%. Second, we had to adapt the breathing exercises to avoid prolonged coughing and oxygen desaturation. Accordingly, we instructed the patient to stop every deep breath before the need to cough and to hold inspiration for better air distribution. In this manner, the patient performed the breathing exercises well and managed to increase his oxygen saturation. Third, the patient had difficulty maintaining sufficient oxygen saturation during physical activity. However, with close monitoring and frequent breaks, he managed to perform strength and walking exercises at a low level without any significant deoxygenation. Exercise progression was low on days 1 to 5, but then increased daily until hospital discharge to a rehabilitation clinic on day 10.

### Case 2: Dyspnea and Anxiety

A 39-year-old man was hospitalized due to an increasingly reduced general health condition, after persistent fever and dry cough for 2 weeks. The patient initially needed 4 L/min of oxygen, had a rapid and shallow breathing pattern at rest and became severely breathless during minor physical activities. In the beginning, physical therapy focused on patient education about dyspnea-relieving positions, the importance of regular mobilization, and deep-breathing exercises. However, it quickly became evident that his anxiety from fear of dying and worries about his future aggravated his dyspnea and vice versa. The patient was so dyspneic, anxious, and weak that he was barely able to walk to the toilet. To counter this vicious circle, the physical therapist actively listened to the patient, explained why he was experiencing breathlessness, and tested suitable positions to relieve his dyspnea. He seemed to benefit from the education and the relaxing breathing exercises, as seen on day 2, when his respiratory rate could be reduced from 30 breaths/min to 22 breaths/min and his oxygen saturation increased from 92% to 96% on 4 L/min oxygen after guiding him through some deep-breathing exercises. Over the next days, his dyspnea and anxiety started to alleviate and he regained his self-confidence. Therapy was progressively shifted to walking and strength training and the patient rapidly advanced to walk 350 m without a walking aid or supplemental oxygen before his discharge home.

### Case 3: Awake Prone Positioning

One week after a positive COVID-19 result this 57-year-old male was admitted to the ICU because of oxygen desaturation (70%) with worsening tachypnea and dyspnea. Physical therapy started immediately after ICU admission. We found a highly dyspneic patient with a high breathing frequency and significant symptom exacerbation from the slightest effort. With hands-on physical therapy guidance, the patient managed to achieve a 135° prone position and to perform deep-breathing exercises resulting in an increase in oxygen saturation from 88% to 96%. Intensive physical therapy and positioning was continued along with 6 to 12 L/min of oxygen therapy over the next days and intubation was avoided. The major challenges in achieving a prone position were the patient’s profoundly reduced respiratory capacity and the high risk of exacerbating his symptoms. However, standard ICU monitoring enabled safe implementation at an individually adapted pace to allow sufficient time for convalescence. After 3 days with this regime, he could be transferred to the normal ward, where physical therapists carried on his rehabilitation with walking and strength training. The patient’s severe instability remained a challenge. Nevertheless, 9 days after ICU admission, the patient was able to leave the hospital as a pedestrian.

### Case 4: Persistent Instability

**Figure 2:**
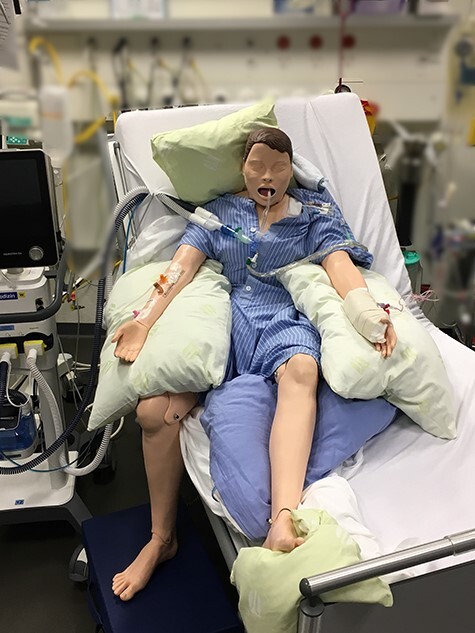
Passive mobilization into a side-position on the edge of the bed (side-edge). To achieve this position, patients are turned to the side and their backs supported with a large pillow (not visible). Then the bedhead is slowly raised, one leg placed on the floor and the position supported with pillows (as shown). The center of gravity remains in the bed, but patients are closely monitored by a nurse or therapist to ensure safety (see also Suppl. Fig. 2).

This 69-year-old male was admitted to the ICU after a dry cough for 2 weeks, oxygenation was poor and computer tomographic imaging showed typical COVID-19 pneumonia. Initially the patient received lung-protective ventilation and targeted sedation, but was otherwise stable. Treatment interventions included passive range of motion and positioning including passive mobilization into a side-edge position (Fig. 2). Over the next days, the patient deteriorated with hemodynamic instability and severe ARDS leading to intermittent prone positioning^10^ and continuous renal replacement therapy. The role of physical therapists during proning was to ensure correct joint positioning and pressure prophylaxis to prevent secondary complications such as nerve lesions,^11^ contractures, or pressure ulcers.^10^ Nevertheless, the long duration and repeated positioning resulted in a small pressure ulcer on the patient’s forehead. After tracheostomy, passive range-of-motion exercises, and passive side-edge mobilization were slowly resumed, whereby asynchronous ventilation and hemodynamic instability remained 2 major problems leading to further sedation and relaxation, thus inhibiting any active participation. After 24 days in the ICU, the patient scored 1/50 points on the Chelsea Critical Care Physical Assessment Tool (CPAx)^12^ and showed severe signs of muscle loss. The patient died soon after withdrawal of life support.

### Case 5: Sputum Clearance

This 57-year-old male was admitted to the ICU with dyspnea, heavy dry cough, and fever 6 days after testing positive for COVID-19. Initially, he was able to exercise and sit in a chair with a physical therapist, but progressive respiratory failure necessitated intubation and proning.^10^ The patient had large amounts of bronchial mucus and required regular suctioning along with respiratory therapy. Secretions were assessed with pulmonary auscultation (presence of crackles)^13^ and by analyzing expiratory flow on the ventilator (sawtooth pattern).^14^ When suctioning failed to improve these clinical signs, 1 to 2 physical therapists used manual airway clearance techniques. The goal of these techniques was to sufficiently increase expiratory flow for effective airway clearance while avoiding alveolar collapse. To achieve this, manual compressions on the chest and abdomen were performed with just enough intensity to modify expiratory flow. After extubation, the patient was still unable to effectively clear his mucus due to weak cough. He continued to need intensive manual airway clearance techniques, nasal rinsing to induce cough and to help expectoration as well as upper and lower airway suctioning. To this end, the patient was treated up to 6 times per day/night. Additional physical therapist interventions included passive range of motion, assisted exercising, and mobilization. At the time of writing, the patient was still in the ICU without ventilatory support.

### Case 6: Dysphagia

This 52-year-old male tested COVID-19 positive 4 days after the beginning of a dry cough, fever, and head and limb pain. One day later, he was hospitalized with exertional dyspnea. He was diagnosed with pneumonia that developed into moderate ARDS needing mechanical ventilation and intermittent dialysis. After extubation, oxygenation was stable with 2 to 3 L/min of oxygen. However, the patient was disoriented and could not communicate verbally. His global weakness (CPAx 11/50) was accompanied by oral and pharyngeal weakness and paresthesia. Spontaneous swallowing frequency and tongue control were severely reduced, and the patient showed insufficient protection from aspiration. This was confirmed by a specialized physical therapist with the Gugging Swallowing Screen, which confirmed severe dysphagia with 2/20 points.^15^ He was treated nil by mouth and received dysphagia therapy such as intensive oral stimulation, facilitation of swallowing, and training of protection mechanisms. After initial agitation and disorientation, the patient started to communicate in single-word phrases, but dysphagia continued to be severe with massive oral and pharyngeal dry saliva residuals that compromised his paresthesia and required regular mouth care. Over the next days, the patient managed to swallow pureéd food and mildly thick fluids under supervision, although cough strength was still weak (Gugging Swallowing Screen 13/20, CPAx 30/50). Nevertheless, he continued to progress and became capable of independent food ingestion (Gugging Swallowing Screen 20/20, CPAx 39/50) before his discharge to a rehabilitation clinic 25 days after admission.

### Case 7: ICU-Acquired Weakness

Paramedics found this 59-year-old female with dyspnea and an oxygenation of 65% on room air and performed immediate tracheal intubation. Moderate ARDS with reduced lung compliance was diagnosed and treated with deep sedation, neuromuscular blocking agents, and prone positioning.

On day 14, a trial of sitting on the edge-of-bed (SOEB) was performed, while she was still intubated and under pressure support ventilation. SOEB required 3 physical therapists to maintain the position, but resulted in a significant increase in her level of consciousness and collaborative state. The next day, she was able to hold her head and sit for about 15 minutes with 2 therapists. Her muscle strength indicated ICU-acquired weakness, with a Medical Research Council sum-score (MRC-SS) of 40/60;^16^ still she continued with small but consistent improvements and started to participate actively in physical therapy sessions. She was encouraged to mobilize herself with exercises against gravity and was actively transferred to a chair each day with the help of 2 physical therapists. She was successfully extubated, but presented postextubation dysphagia. The physical therapy team closely monitored her for secretion management and cough stimulation and continued her physical rehabilitation. On day 19, she started to walk with a walking aid, although at this point oxygen desaturation during exercise training became evident (89% with 3 L/min of oxygen). After 25 days, she was transferred to the institution’s rehabilitation facilities, where a battery of tests indicated persistent physical function impairment (MRC-SS 52/60, physical function ICU test score^17^ 9/12, Timed Up & Go 23 seconds, short physical performance battery 4/12).

### Case 8: Delirium

This 33-year-old female patient had typical COVID-19 symptoms such as high fever, dry cough, headache, and dyspnea about 1 week before ICU admission. She was intubated and proned due to rapid respiratory deterioration. For the following 6 days, her situation was unstable, and physical therapy consisted of prone positioning and prevention of secondary damage. From day 7 onwards, she started to improve rapidly and could be mobilized passively into a side-edge position. After extubation, she presented postextubation dysphagia and severe ICU-acquired weakness (MRC-SS 36/60). She also suffered from pronounced delirium and anxiety and said repeatedly that she had been abducted and that she believed she had to die. She seemed to feel threatened by us and it was difficult to calm her down. Due to the pandemic measures of the Swiss government, hospital visits were not generally allowed, but because her anxiety was limiting her rehabilitation, her husband was granted an exceptional permission to visit her. This seemed to give the patient a short sense of security, and she started to participate in some basic functional activities (CPAx 21/50). Nevertheless, the delirium did not resolve upon her transfer to a peripheral acute hospital.

### Case 9: Neurological Complications

This 66-year-old male patient was admitted to the hospital due to an ischemic left-hemispheric stroke in addition to a dry cough and fever. He tested positive for SARS-CoV-2 the following day but continued to deteriorate resulting in severe ARDS, intubation, and ICU admission. Despite repeated proning,^10^ gas exchange did not improve sufficiently and the patient was placed on veno-venous extracorporeal membrane oxygenation for 7 days. After sedation was stopped, the patient continued to be somnolent and unable to communicate or to follow commands. Physical therapy therefore focused on perception training, movement exercises, airway-clearing techniques, dysphagia therapy, and mobilization. A first SOEB trial had to be discontinued due to hemodynamic instability. Instead, the patient was positioned in a side-edge position (Fig. 2), which he tolerated better and where an intensive exercise training including trunk and head control was conducted. Nevertheless, muscle tone and strength remained severely reduced, particularly on his hemiplegic side, and a second SOEB trial failed again. Physical therapy was also limited because of reduced self-activity and suspected impaired perception and visual acuity. Consequently, occupational therapy was involved to create a basis of communication, to support functional initiation of upper limb movements, and to integrate perception-training into activities of daily living. Currently, the patient tolerates spontaneous breathing trials, shows signs of being alert during therapy, but cannot communicate. He is hemodynamically stable, even in an SOEB position, but remains functionally dependent (CPAx 6/50).

### Case 10: Difficult Weaning

**Figure 3:**
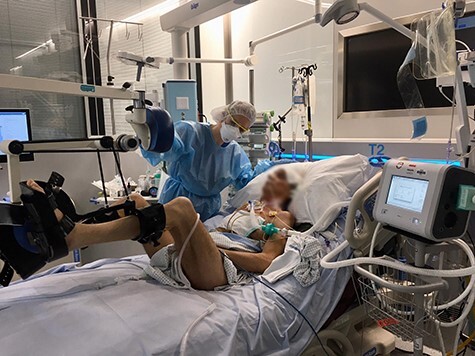
A patient under assisted ventilation starts to move again by cycling actively in bed at a moderate workload (MOTOmed letto2; Reck-Technik GmbH, Betzenweiler, Germany). Reproduced with written informed patient consent.

A 66-year-old male started to present symptoms of fever, dyspnea, coughing, asthenia, lack of appetite, nausea, and vomiting. He was admitted to the acute care unit for observation and oxygen therapy, but his oxygen requirements constantly increased due to moderate ARDS. After 12 days of deep sedation, neuromuscular blocking agents, and proning with daily passive range of motion, the patient finally started to initiate active movements and was passively transferred to a chair. However, due to a persisting difficult weaning status,^18^ probably related to respiratory muscle weakness, tracheostomy was performed [ventilator settings: pressure support 10 cmH_2_O, positive end-expiratory pressure (PEEP) 8 cmH_2_O]. Subsequently, the patient showed significant improvement in his physical functions with active SOEB, chair-transfer with the help of 2 physical therapists, and active in-bed cycling against resistance for 20 minutes (Fig. 3). The strategy was to increase pressure support (by 5 cmH_2_O) during efforts to reinforce exercise training effects, unloading respiratory muscles. This strategy along with a highly collaborative patient culminated in his rapid improvement in physical function (MRC-SS 58/60, physical function ICU test score 10/12, walking distance 10 m), although he was still experiencing fatigue, inspiratory muscle weakness (maximal inspiratory pressure of −45 cmH_2_O),^19^ and dysphagia upon his transfer to a step-down unit.

### Case 11: Telerehabilitation

This 77-year-old male patient was transferred to our ICU 1 week after his COVID-19 diagnosis due to continuing respiratory decompensation requiring intubation. Following the acute phase, with intermittent proning, the patient continued to be hemodynamically unstable and was difficult to wean. Rehabilitation proved challenging under these conditions, and physical therapists had to reevaluate and adapt their interventions daily according to his condition. After 2 weeks, he was tracheotomized and started to improve very slowly. One week after tracheostomy, the patient was able to speak for the first time after a cuff-down trial and with the help of a speaking valve. But the patient spoke only a few words with us and it was often difficult to involve him in exercises. Two days later, he was able to communicate with his relatives via video telephony. This was a very emotional moment for everyone involved, but it improved his communication and he was able to express to his wife that he had no strength left to continue. However, through the family’s active participation in his early rehabilitation process, they were able to reinforce his confidence and motivation. He was discharged to a rehabilitation clinic severely weak (MRC-SS 40/60) and functionally impaired (CPAx 22/50), but continued to progress in slow steps.

## Discussion

This case series describes the numerous indications and treatment interventions for physical therapy and reveals severe post-COVID complications with a slow and fluctuating recovery of hospitalized patients with COVID-19 in Switzerland. It highlights challenges in the treatment of these patients and underlines the necessity for repeated assessment and clinical reasoning. Given that COVID-19 is a new illness, this real-life evidence can be helpful to advance knowledge and inspire new research. We especially recommend investigating interventions like awake prone positioning and early breathing exercises on acute care hospital wards as well as developing and evaluating post-COVID rehabilitation programs.

Cases 1 to 3 primarily illustrate the high instability of oxygenation, the link between anxiety and dyspnea, and rapid deconditioning in hospitalized patients with a moderate COVID-19 disease. In accordance with recently published evidence we found that awake proning increased oxygenation,^20^ but had to be closely guided to avoid desaturation or discomfort. Similarly, other exercise interventions had to be individually adapted and often required a very slow progression with close monitoring of oxygenation and signs of dyspnea. The therapy environment was further complicated by the patients’ anxiety and isolation. Physical therapists were therefore called upon to provide emotional support and comfort to these distressed patients, while at the same time delivering therapy in the form of individualized dyspnea and respiratory management, patient education, and supervised exercises.

Cases 4 to 11 represent patients who were mechanically ventilated and critically ill with moderate to severe ARDS due to SARS-CoV-2. Physical therapists were generally involved within 48 hours of ICU admission and usually provided 1 to 2 daily treatments. They assisted in early ICU management such as prone positioning or respiratory care and initiated early mobilization and rehabilitation. In a recent point-prevalence study, Switzerland displayed one of the highest rates (33%) of active, out-of-bed mobilizations in patients on mechanical ventilators compared with other countries like the United States (16%) or Germany (24%).^9^ Despite this culture of early mobility, patients with COVID-19 were mobilized relatively late during their ICU stay. The main reasons were deep sedation and pulmonary/hemodynamic instability. To overcome these barriers, mobility was initiated slowly and repeatedly. This included the use of in-bed cycling or the progression of a side-edge position into a full SOEB. Nevertheless, we saw profound weakness and functional impairment upon awakening that often required several therapists for a SOEB or chair transfer. Many patients also experienced postextubation dysphagia that required intensive dysphagia therapy including respiratory techniques to avoid aspiration. Dysphagia is common after prolonged intubation, and multidisciplinary management, including routine screening, is highly recommended.^21^^,^^22^

Many of the featured complications like ICU-acquired weakness, weaning failure, postextubation dysphagia, delirium, or anxiety have been previously described.^21^^,^^23^ We cannot exclude that these symptoms are a specific complication of SARS-CoV-2. Even so, we recommend using current guidelines like the awakening and breathing coordination, delirium monitoring/management, early exercise/mobility, and family engagement/empowerment (ABCDEF) bundle, which aims to reduce these complications through interprofessional collaboration.^24^^,^^25^

Cases 8 and 11 both illustrate the importance of family visits for patients’ reorientation and recovery. Family presence is considered an important resource in the rehabilitation process, but might not always be feasible during a pandemic. In our experience, the use of technology such as video calls during physical therapy proved particularly helpful for patients’ reorientation and rehabilitation progress. Alternatively, delirium can be managed with nonpharmacological interventions such as mobilization, person-centered care including regular reorientation and reassurance, or simple earplugs to enhance sleep.^26^ We and others^26^ therefore advocate that evidence-based interventions such as the ABCDEF bundle be continued with high priority to meet the multiple psychological, cognitive, and physical complications of critical illness as early as possible.

### Limitations

This case report has limitations. Patients were included retrospectively and were highly selected to represent common aspects of physical therapy care during the COVID-19 pandemic. We describe a fairly representative cohort of patients with COVID-19. Nevertheless, interventions cannot be generalized, nor does this report prove their efficacy. Data collection was somewhat limited by the pandemic environment and the use of different outcome measures between hospitals. Finally, this report does not provide any data on long-term outcomes and thus only mirrors the acute phase of COVID-19.

## Conclusion

Hospitalized patients with COVID-19 can experience different disease courses and numerous symptoms, which, besides pulmonary and hemodynamic instability, include anxiety, dyspnea, sputum, ICU-acquired weakness, delirium and postextubation dysphagia. These symptoms were often highly unstable and had to be closely monitored during physical therapy to allow a safe implementation. Active rehabilitation started relatively late in the ICU course, but as early as possible due to daily screening. We suggest that all hospitalized patients with COVID-19 should be assessed regularly by a physical therapist to evaluate indications and to use clinical reasoning and previously established evidence-based methods for their treatment.

## Supplementary Material

Supplementary_material_pzaa194
