# Supplementary material for: Early Physical Therapist Interventions for Patients With COVID-19 in the Acute Care Hospital: A Case Report Series
Source: Phys Ther. 2020 Oct 19;101(1):pzaa194. doi: 10.1093/ptj/pzaa194 (PMC7665777; doi:10.1093/ptj/pzaa194)
Supplement: Supplementary_material_pzaa194 [file supplementary_material_pzaa194.pdf]

Supplementary material

Early physical therapy interventions for COVID-19 patients in the acute care hospital: a case report series

Sabrina Eggmann[1], Angela Kindler[1], Andrea Perren[1], Natalie Ott[2], Frauke Johannes[3], Rahel Vollenweider[3], Théophile Balma[4], Claire Bennett[5], Ivo Neto Silva[5], Stephan M. Jakob[6]

[1] Department of Physiotherapy, Insel Group, Inselspital, Bern University Hospital, Bern, Switzerland
[2] Institute of Therapies and Rehabilitation, Kantonsspital Winterthur, Winterthur, Switzerland
[3] Department of Physiotherapy and Occupational Therapy, University Hospital Zurich, Zurich, Switzerland
[4] Department of surgery and anesthesia, cardio-respiratory physiotherapy, Lausanne University Hospital, Lausanne, Switzerland
[5] Intensive Care Unit, Department of Acute Care Medicine, Geneva University Hospitals (HUG), Geneva, Switzerland
[6] Department of Intensive Care Medicine, Inselspital, Bern University Hospital, University of Bern, Bern, Switzerland

Methods

Settings

All five hospitals were highly involved in the primary care of COVID-19 patients. These hospitals are representative of a large part of Switzerland and include the German and French speaking regions. Further information on hospitals and the physical therapist services is presented in the supplemental S1 Table. We did not allocate the names of the treating hospital to individual cases to ensure the anonymity of our patients.

Participant selection

This case report series aims to describe the wide spectrum of patient presentations as well as the large range of physical therapy interventions in order to illustrate the extensive role of physical therapists during the pandemic. Cases were therefore selected to represent this variety and have been titled accordingly.
In a first step, the first author (SE) contacted physical therapists from three other university hospitals and one large regional hospital to discuss their participation in this project. Subsequently, each hospital screened their current and past case load and had selected a few suitable candidates by the 27th of April 2020. These potential cases were then discussed anonymously by phone or e-mail with the first author to discuss their eligibility. To limit repeated symptom presentation or therapy interventions, the ultimate decision to include a patient was made by the first author (SE).

Data collection

All cases were included retrospectively, whereby the majority were hospitalized patients at the time of recruitment. Routine hospital data and written informed consent were collected by the treating physical therapist. The data was collected over the four-week period from 20th April 2020 to 18th May 2020.

S1 Table. Information about the five hospitals and their physical therapy services.

	Hospital A	Hospital B	Hospital C	Hospital D	Hospital E
**Type of hospital, number of beds**	University hospital<br>>900 beds	University hospital,<br>>1000 beds	University hospital,<br>>800 beds	County hospital,<br>500 beds	University hospital,<br>>1000 beds
**Number of COVID-19 patients**	ICU: 46	Acute ward: 517<br>ICU: 123	ICU and acute ward: 147	Acute ward: 78<br>ICU: 13	ICU: >140 cases
**Multi-disciplinary team and communication**	**Team** generally includes nurses, medical doctors, PTs and OTs. Other services available upon requests.<br>**Communication:** daily morning rounds with evaluation of every patient.<br>Multidisciplinary team visit with pharmacist twice/week.	**Team** generally includes nurses, medical doctors, PTs and OTs. Other services available upon requests.<br>**Communication:** daily morning rounds with evaluation for start of mobilization.<br>Multidisciplinary team visit with pharmacist twice/week.	**Team** generally includes nurses, medical doctors and PTs. Other services available upon requests.<br>**Communication (ICU):** daily morning rounds with evaluation for start of mobilization.<br>Multidisciplinary team visit with pharmacist twice/week.<br>**Communication (wards):** Daily exchange with responsible nurse and (if required) physician about recovery trajectory, aims and proceeding.	**Team** includes nurses, medical doctors and PTs. Other services are available upon request.<br>**Communication (ICU):** Daily morning rounds with nurses to discuss daily schedule and evaluate mobilization. Regular exchange with physicians when needed.<br>**Communication (wards):** Daily exchange with responsible nurse and (if required) physician about recovery trajectory, aims and proceeding.	**Team**: Includes nurses, assistant nurses, medical doctors, PTs, OTs, dietician, pharmacist. Other services available upon request (speech therapist).<br>**Communication**: daily morning rounds with evaluation of every patient.

**PT service hours (usual, pandemic)**	**Usual:** 7am to 10pm, 7 days per week<br>**Pandemic:** 7am to 11pm, 7 days per week	**Usual:** 24 hours, with 1 PT overnight, 7 days per week<br>**Pandemic:** 24 hours, with 2 PTs overnight, 7 days per week	**Usual:** 8am to 5pm, reduced weekend services<br>**Pandemic:** 8am to 11pm, 7 days per week	**Usual/pandemic:** 8am to 5pm, reduced weekend service	**Usual:** 7am to 9:30pm, 7 days per week<br>**Pandemic:** 24h/24, 7 days per week
**Years of experience in the acute hospital/ICU of the treating PT**	1 to 20 years of experience in ICU	1 to 15 years of experience in the acute hospital and ICU	5 to 15 years of experience in the acute hospital and ICU	6 to 10 years of experience in the acute hospital and ICU	**Senior team**: 2 to 10 years of experience in the ICU<br>**Junior team**: a few weeks to a few months experience. They work under the supervision of the senior team. This solution is one among others which were created to reinforce acute physical therapy teams in order to face the COVID-19 outbreak
**PT referral process**	**ICU:** institutional standing orders: PT screening of all patients and treatment according to patient's risk. Mobilization level prescribed by medical doctor.	**ICU:** institutional standing orders: PT screen all patients and treat by risk. Mobilization level is order by medical doctor.<br>**General wards:** referral by treating physician, PT interventions are based upon PT clinical reasoning.	**ICU:** institutional standing orders: PT screening of all patients and treatment according to patient's risk. Mobilization level prescribed by a medical doctor.<br>**General wards:** referral by treating physician, PT interventions are based upon PT's discretion.	**ICU (usual and pandemic):** Referral by treating physician, mobilization level is ordered by medical doctor, interventions are based upon PT clinical reasoning.<br>**General ward (usual):** Referral by treating physician, interventions are based upon PT clinical reasoning.<br>**General ward (pandemic):** Daily screening of all COVID-19 patients for therapy needs by a PT (institutional standing orders), eligible patients were discussed with treating physician as needed. Interventions were based upon PT clinical reasoning	**ICU**: ICU standing order. PT screening of all patients and treatment according to patient's risk. Multidisciplinary discussions are normally undertaken to clarify/resolve perceived contraindication or barriers to early mobilization.<br>**Intermediate Units and General wards:** referral by treating physician, PT interventions are based upon PT's discretion.

**Implementation of prone positioning**	**ICU:** prone positioning was medically led and initiated. PTs assisted in turning maneuvers and were responsible for correct positioning.	Prone positioning was medically led and initiated. PTs assisted in turning maneuvers and were responsible for correct positioning (daytime: specialized proning team including PT). OT responsible for awake proning.	**ICU:** prone positioning was medically led and initiated. PTs assisted in turning maneuvers and were responsible for correct positioning. Awake proning in non-intubated patients was led by PT following medical consultation.<br>**General wards:** prone positioning was initiated and performed by PTs. If feasible, PT instructed nurses and patients in awake proning.	**ICU:** prone positioning was medically led and initiated. PTs assisted in turning maneuvers and supported correct positioning, when available, otherwise nurses performed the turning maneuvers and positioning<br>**General wards:** prone positioning was initiated and performed by PTs. If feasible, PT instructed nurses and patients in awake proning.	**ICU:** prone positioning was medically led and initiated. PTs assisted in turning maneuvers and were responsible for correct positioning.<br><br>**Intermediate Units:** Self-proning strategy was led by PT following medical consultation and instructed to nurses and patients.
**Extended PT roles (usual, pandemic)**	**Usual/pandemic**: dysphagia therapy, management of tracheal cannula	**Usual/pandemic:** tracheostomy management, suctioning, dysphagia therapy, involved in patient diary and family communication	**Usual/pandemic:** tracheostomy management, suctioning, dysphagia therapy, involved in patient diary and family communication	**ICU usual/pandemic:** suctioning, dysphagia therapy<br>**General wards pandemic:** Screening of eligible patients for PT	**Usual/pandemic**: manual and mechanical airway clearance techniques, suctioning, tracheostomy management, assistance in bronchoscopies procedure, performance of protected mini-bronchoalveolar lavage, responsible for the application of noninvasive-ventilation (NIV) therapies (including the emergency department)

Abbreviations: PT = physical therapist, OT = occupational therapist

S1 Figure. Simulated 135° prone position.

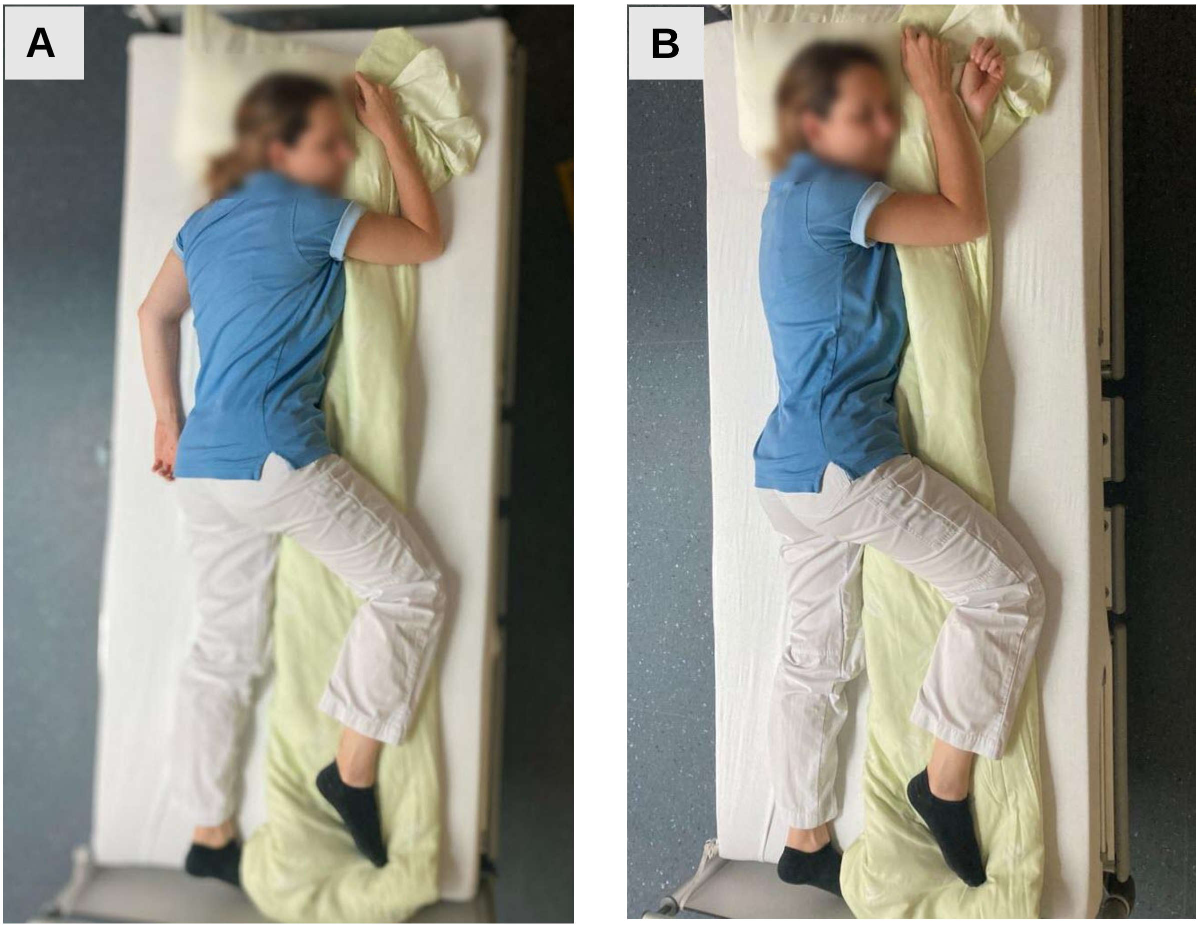

Similar to a full prone position the 135° position aims to increase oxygenation by recruiting the dependent (dorsal) lung thus increasing the ventilation-perfusion ratio. It is especially useful if one lung is better than the other (good lung down principle) or for patients who do not tolerate the full prone position.

A: The 135° prone position was achieved for unconscious patients by sliding them to the edge of the bed, turning them to their side over their adducted arm and positioning them with several pillows as illustrated. The position was maintained for several hours similar to the full prone position. The maneuver requires about 5 helpers in mechanically ventilated, sedated patients.

B: Awake patients were slowly guided through the same process step-by-step based on their abilities and with breaks as needed to avoid overexertion. Patients often spontaneously chose a position with both arms placed in front of them. The position was maintained as long as comfortable for the patient, usually between 30 to 90 minutes. The maneuver usually requires 1-2 helpers to secure lines and installations and to help with positioning.

S2 Figure. Simulated side-edge position.

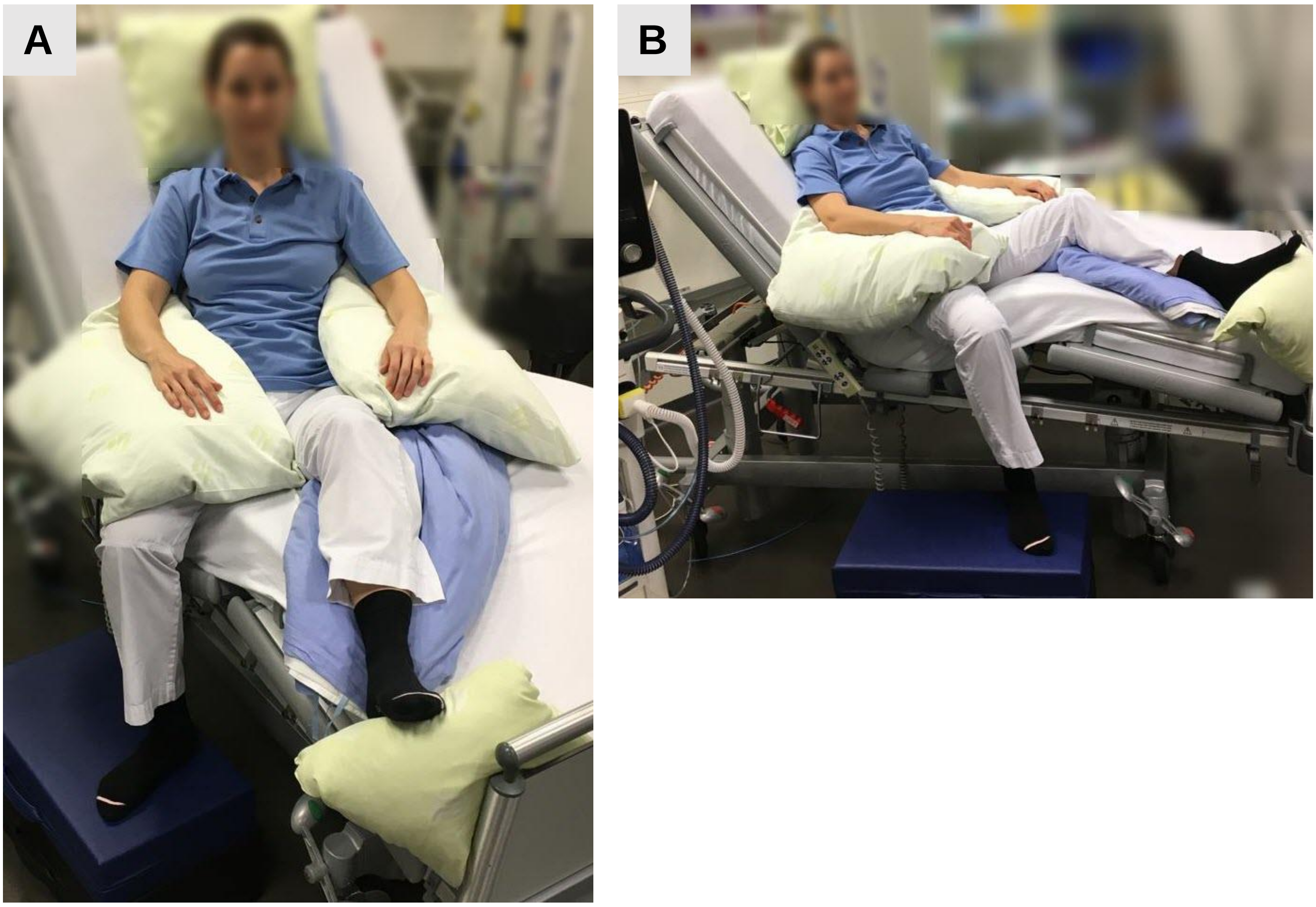

A side-edge position can usually be achieved with 1-2 helpers. Sitting duration depends upon patient’s physiological stability and tolerance but usually ranges between 5 to 60min. The position aims to facilitate awakening, trunk and head control or may be used as a middle step before a full mobilization to the edge of the bed.

A: Front view of the side-edge position.

B: Side view of the side-edge position.
